# Arthroscopic Treatment of Intraosseous Ganglion Cysts of the Carpus

**DOI:** 10.3389/fsurg.2022.798432

**Published:** 2022-06-07

**Authors:** Xiao-Feng Teng, Xin-Kun He, Hong Chen, Jian Ruan

**Affiliations:** Department of Hand Surgery, Ningbo No.6 Hospital, Ningbo, China

**Keywords:** waist, arthroscopy, intraosseous ganglion cyst, bone implantation, treatment

## Abstract

**Objective:**

To investigate the application and the clinical efficacy of wrist arthroscopy in the treatment of carpal intraosseous ganglion cysts (IGCs).

**Methods:**

A retrospective case study method was adopted to analyze the clinical data of 28 patients with carpal IGCs admitted to the Sixth Hospital of Ningbo from April 2012 to January 2019. A hypodensity in the bone was shown by X-ray before the operation, with hypodensity and cystic change in the bone being confirmed by computed tomography and magnetic resonance imaging. Arthroscopic open window of the wrist, cystectomy, and autologous iliac bone graft implantation were conducted. Regular postoperative X-ray combined with CT follow-ups were conducted to observe the healing after bone implantation. Patients were followed up regularly and assessed by the Modified Mayo Wrist Score in four aspects of the postoperative pain, wrist mobility, grip, and function to provide an objective overall assessment of the therapeutic outcome.

**Results:**

All 28 patients were followed up for 8–16 months, with an average follow-up duration of 10 months. After the operation, pain disappeared completely for 25 patients, and 3 cases showed significant improvement. All cases were pathologically confirmed as ganglion cysts and had first-stage bony healing after bone grafting with an average healing time of 10.8 weeks. The grip returned to normal for all patients, and wrist flexion and extension were the same as the healthy wrist for 25 patients, with a Modified Mayo Wrist Score of excellent in 19 cases and good in 9 cases. No recurrence was observed.

**Conclusion:**

In patients with symptomatic carpal IGCs, the application of arthroscopic open window, cystectomy, and autologous bone graft implantation could achieve satisfactory clinical therapeutic effects.

## Introduction

An intraosseous ganglion cyst (IGC) is a benign non-neoplastic bone lesion that is histologically similar to a soft tissue ganglion cyst and contains a mucus-like substance without epithelial cells or a synovial layer (1). IGC is the most common bone disease that mainly occurs in the lunate and scaphoid bone (2). IGC is classified into two types. Type I is idiopathic, the pathogenesis of which is still unclear. Trauma, herniation of the joint capsule, mucinous degeneration, intramedullary epithelial metaplasia of mesenchymal cells, and congenital defects in synovium-producing cells have been associated with the development of IGC. Type II is the penetrating type, caused by the invasion of proximal cortical material (usually a ganglion cyst in the dorsal soft tissue) into the cancellous bone cavity (1, 3).

Generally, patients present with chronic wrist discomfort and pain or no symptoms at all. IGC is first detected by an X-ray of the wrist, which shows intraosseous hypodense changes. Computed tomography (CT) and magnetic resonance imaging (MRI) of the wrist can further clarify the diagnosis. The differential diagnosis of IGC includes osteoarthritic cyst, post-traumatic cyst, simple bone cyst, and aneurysmal bone cyst. Kienbock’s disease, osteoid osteoma, osteoblastoma, and degenerative cysts (as in rheumatoid arthritis) should also be included in the differential diagnosis when symptoms manifest as wrist pain (3, 4).

Treatment options depend on clinical and imaging findings. Surgery is required if the IGC is symptomatic or if imaging data suggest an increase in size (5). The growing IGC can lead to traumatic and collapsed fractures with serious complications (6). When the IGC has completely stopped growing, and there is no cortical defect or collapsed fracture, regular imaging follow-up is recommended. CT has a special role in the decision-making process. The progressive aggravation of local pain at the wrist suggests the need for surgery (6, 7). Surgical treatment of IGC includes cystectomy, drilling, burring, scraping, saline injection, and filling of cancellous bone (8). Depending on the location of the cyst, an anterior approach, dorsal approach, or other surgical methods can be selected, such as lesion osteotomy and radial wrist joint fusion (4, 9).

Wrist arthroscopy is a minimally invasive surgical technique, usually performed under local or regional anesthesia, that reduces the intra-articular surgical area, thus minimizing the incidence of postoperative stiffness. Arthroscopy has been widely applied for the treatment of wrist diseases, such as in the treatment of scaphoid fracture, distal radius fracture, scapholunate ligament repair, and ganglion cystectomy of soft tissues in the wrist (10–12). There are few reports on the application of arthroscopy for carpal IGCs in the wrist. Alexandre Cerlier, Jr. et al (12). conducted arthroscopic cystectomy and bone grafting in four patients with carpal IGCs. The pain had disappeared in all patients within 2 months after surgery, the average grip was restored almost fully, and the motion range of the joint was essentially the same as that of the healthy wrist. There were no complications after surgery. Therefore, it was believed that arthroscopic treatment of carpal IGC might be more helpful in overcoming the shortcomings of traditional open surgery in terms of complications. N Borisch et al (13). applied arthroscopic ganglion cystectomy in 88 patients with a total of 92 wrists between 2007 and 2010, which achieved a 90% patient satisfaction rate. The only complication was the development of complex regional pain syndrome in one patient, with a recurrence rate of 12.5%. This study aimed to investigate the application and the clinical efficacy of wrist arthroscopy in the treatment of carpal intraosseous ganglion cysts (IGCs).

## Materials and Methods

### General Data

A retrospective case study method was adopted to analyze the clinical data of 28 patients with carpal IGCs admitted to the Sixth Hospital of Ningbo from April 2012 to January 2019. There were 6 males and 22 females, with ages between 26–38 years and an average age of 31 years. All patients had varying degrees of wrist discomfort and pain, along with decreased grip strength, and no significant movement restriction of the wrist. There were 16 cases of the right wrist and 12 cases of the left wrist. There were 17 cases involving the lunate, 8 cases of the triquetrum, and 3 cases of the scaphoid bone. The duration from the onset of the disease was 6 to 23 months. Well-defined hypodensities were visible in the frontal and lateral radiographs of the wrist, together with CT and MRI examinations, and one case with lunate cortical rupture. This study was conducted in accordance with the declaration of Helsinki and approved by the Ethics Committee of Ningbo No.6 Hospital. Written informed consent was obtained from all participants.

### Surgical Methods

The indications for operative treatment include pain, limited function of the wrist, lesion growth, and/or suspicion of malignancy based on diagnostic tests. Brachial plexus anesthesia was routinely conducted, and the patient was placed in the supine position and the upper arm was tied with a balloon tourniquet with a pressure of approximately 250 mmHg for hemostasis. The operative upper extremity was placed in a Linvatec wrist traction tower and 10–12 lbs of traction were applied through the index, middle, and ring fingers. Flushing fluid was filled by the gravity perfusion system. The radial and ulnar midcarpal portals were accessed through small incisions in the dorsal wrist using a 2.7 mm arthroscope. In all cases, arthroscopic examination of the midcarpal joint space was conducted, and no inflammatory synovial membrane was observed. In one case, the bone cyst broke out from the space between the scaphoid bone and lunate, and the rest of the carpal cortex was intact. The observation access and operation access were established in the midcarpal joint space. CT scan was used to determine the location of the cyst from pre-operative imaging. An arthroscopic grinding drill was adopted to conduct an open window on the dorsal surface of the carpal bone, the area of the intraosseous cyst was probed, and light clear fluid was visible. The wall of the cyst was scraped completely with a suitably sized spatula and sent for pathological examination (see Supplementary Figure S1). First stage bone grafting was performed in all of the cases. Autologous cancellous iliac bone was taken, cut into rice-sized pieces with scissors, and implanted into the carpal defect through the sheath. The normal saline in the articular cavity was drained and turned into anhydrous articular cavity, and then butyl was injected through the syringe needle α- Cyanoacrylate was injected into the bone surface of bone graft for fixation to prevent bone detachment. Then the skin was sutured.

### Postoperative Treatment and Follow-up

After the operation, a regular short arm plaster cast was adopted for immobilization. The active motions of the metacarpophalangeal joint and each interphalangeal joint were started on the second day after surgery, and the stitches and cast were removed 2 weeks after surgery. Then active functional exercises of wrist flexion and extension were conducted. After the fracture healed, with confirmation of X-ray combined with CT, the patient resumed daily activities and work. The Modified Mayo Wrist Score was adopted during the follow-up to make an objective overall assessment of the therapeutic effects by evaluating the following four aspects: postoperative pain, wrist mobility, grip, and function. A score of 91–100 points was considered excellent, 80–90 points was considered good, 65–79 points was considered acceptable, and fewer than 65 points was considered poor.

## Results

**Figure 1:**
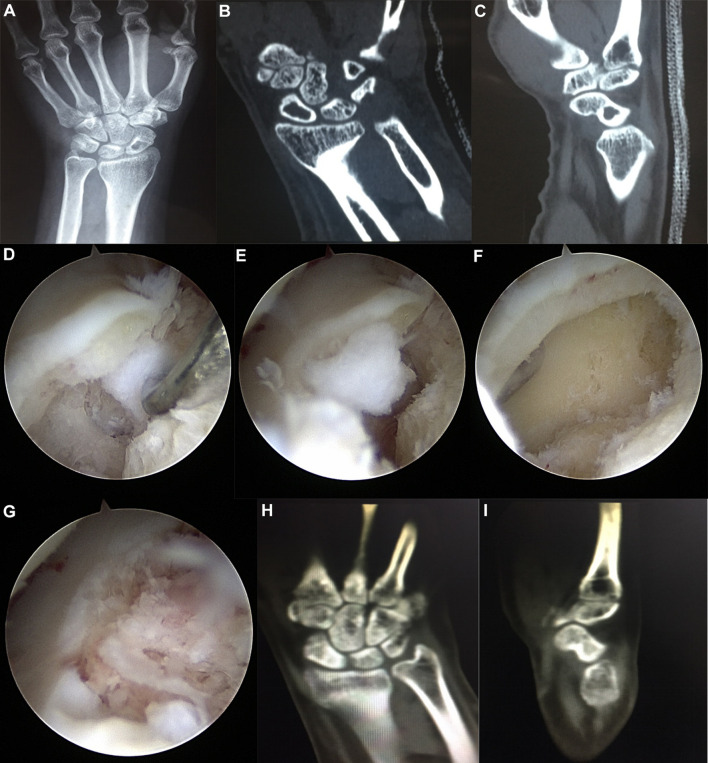
A typical case. (**A**) pre-operative X-ray; (**B**) pre-operative coronal CT; (**C**) pre-operative sagittal CT; (**D**) Intraoperative fenestration of scaphoid bone showed cyst; (**E**) Scaphoid cyst scraped out during operation; (**F**) Navicular cavity after curettage of cyst during operation; (**G**) Cavity bone grafting after curettage of cyst during operation; (**H**) The coronal CT scan showed bone healing in the scaphoid bone graft area after 3 months of follow-up; (**I**) The sagittal CT scan showed bone healing in the scaphoid bone graft area after 3 months of follow-up.

**Figure 2:**
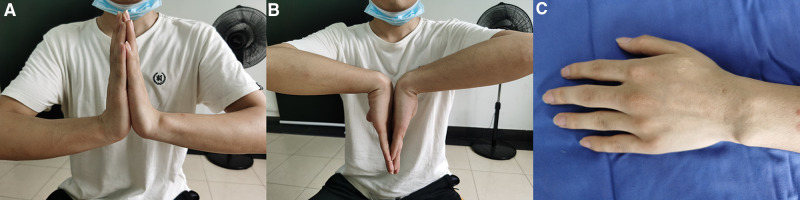
Postoperative follow-up of the case. (**A,B**) Postoperative functional recovery; (**C**) Postoperative wound recovery.

All 28 patients were followed up for 8 to 16 months, with an average follow-up duration of 10 months. All cases were pathologically confirmed as ganglion cysts and had first-stage bony healing after bone grafting with an average time of 10.8 weeks. The wound at the iliac bone site healed well, the scar was small, with an average of about 2 cm, and there was no pain or skin numbness at the wound of wrist. The pain at wrist disappeared in 25 cases and improved significantly in 3 cases. The grip returned to normal in all patients. In 25 cases, the wrist flexion and extension were the same as that of the healthy wrist, and in 3 cases, it was 85%–90% of the healthy wrist. The results of the Modified Mayo Wrist Scores showed excellent in 19 cases and good in 9 cases. No recurrence was observed. The typical case was shown in Figure 1. Postoperative follow-up of the case was shown in Figure 2.

## Discussion

IGC is a benign bone lesion that primarily involves the metaphysis and epiphyseal region of long bones. It can also be found in short bones such as the wrist. Ganglion cysts can occur at any age but are most common in patients aged 20–50 years. The lunate is the most common site of ganglion cysts at the wrist (14). The complications associated with this lesion (mainly the risk of pathological fracture) can be severe and even can lead to irreversible injury (15). Therefore, early diagnosis and intervention are necessary. The typical X-ray manifestation is a cystic hypodensity within the wrist with well-defined borders and a sclerotic rim (16). CT, MRI, and special bone scan examinations can help to make a definite and differential diagnosis, and also have reference significance for the preoperative localization, selection of surgical approach, and selection of the open window site of cystectomy (17, 18).

### Surgery Indications

Carpal IGCs are not uncommon and are usually found in the dorsal carpal, but may also be found in the metacarpals, carpals, and even in interphalangeal joints. In adults, IGCs are more common on the dorsal side of the wrist, while in children, they are more common on the palmar side (13). Most carpal IGCs are asymptomatic, and once symptoms appear, they can affect activities of daily living. Carpal IGC mainly manifests as wrist soreness and discomfort or pain, together with decreased wrist mobility, decreased hand grip, and in severe cases, abnormal sensation or wrist dysfunction (19). Therefore, surgical intervention is recommended for those patients who have symptoms or in whom the imaging data suggest growing cysts (5). The aim of surgery is to reduce local pain, improve wrist function, and prevent complications.

### Options for the Surgical Methods

The traditional surgical treatment is mainly open surgery, which involves cystectomy of the carpal IGC, and bone grafting with autogenous or artificial bone. There is a risk of damaging the intercarpal ligament during the operation. The main complications are joint stiffness and vascular disorders, and there may also be postoperative wrist pain or swelling of the hand (20). Recently, wrist arthroscopy has been adopted in clinical practice. This technique causes little injury to the wrist and allows patients to begin functional wrist exercises early after surgery. Moreover, the bone grafting with autogenous or artificial bone should be used during the operation, in order to accelerate the growth of bone and avoid fracture after postoperative activities. In this study, the iliac crest was used as bone graft due to the better concealment Additionally, the use of Lister tubercle and olecranon bone graft also are alternatives for the donor site. Although, the Lister tubercle is not been widely accepted as a bone graft for Chinese people.

In the present study, all 28 patients were operated under wrist arthroscopy and compared with traditional open surgery. The postoperative recovery period was shorter, and the functional recovery was ideal. Three cases still had mild postoperative pain, one of which was caused by the sensory nerve injury during surgical incision and improved after treatment. Although the patient still had discomfort, it differed from the cause of preoperative pain. Mild postoperative pain in two patients was considered to result from the bone opening on the dorsal side of the wrist and was caused by the scar proliferation reaction after healing, which was also not the same cause as the preoperative pain.

### Precautions for Operation

In the authors’ opinion, the following aspects should be noted during the surgical operation: (1) The midcarpal joint is a narrow, curved joint, which is difficult to operate on arthroscopically, so it was important to establish good access for observation and operation. In the present study, the intraosseous ganglion cyst of the scaphoid bone was observed through the ulnar midcarpal space and operated on through the radial midcarpal space, and vice versa for the intraosseous ganglion cyst of the lunate and the triquetrum; (2) Positioning of the carpal opening was important to minimize the damage to the non-cystic area of the carpal bone. In the present study, the preoperative coronal and sagittal CT images were combined to initially find the intraosseous ganglion cyst, and the intraoperative vision could clarify the location of the open window in the wrist; (3) Since the proximal and distal carpal bones are articulated structures, it was important to minimize the area of articular surface injury. In the present study, the open window was positioned in the dorsal non-articular surface area of the carpal bone to avoid damage to the articular cartilage, avoiding the occurrence of secondary localized carpal arthritis; (4) After autologous bone graft implantation in the wrist, a medical adhesive of butyl α-cyanoacrylate was applied to the surface of the bone graft area to prevent the bone graft particles from dislodging.

In patients with symptomatic carpal IGCs, the application of arthroscopic open window, cystectomy and autologous bone graft implantation gives satisfactory clinical therapeutic effects.

## Data Availability

The original contributions presented in the study are included in the article/Supplementary Material, further inquiries can be directed to the corresponding author/s.
